# The Dynamic Counterbalance of RAC1‐YAP/OB‐Cadherin Coordinates Tissue Spreading with Stem Cell Fate Patterning

**DOI:** 10.1002/advs.202004000

**Published:** 2021-03-08

**Authors:** Shengjie Jiang, Hui Li, Qiang Zeng, Zuohui Xiao, Xuehui Zhang, Mingming Xu, Ying He, Yan Wei, Xuliang Deng

**Affiliations:** ^1^ Beijing Laboratory of Biomedical Materials, Department of Geriatric Dentistry Peking University School and Hospital of Stomatology Beijing 100081 P. R. China; ^2^ School of Systems Science Beijing Normal University Beijing 100875 P. R. China; ^3^ Beijing National Laboratory for Condensed Matter Physics and Laboratory of Soft Matter Physics Institute of Physics Chinese Academy of Sciences Beijing 100190 P. R. China; ^4^ Department of Dental Materials & Dental Medical Devices Testing Center National Engineering Laboratory for Digital and Material Technology of Stomatology Peking University School and Hospital of Stomatology Beijing 100081 P. R. China

**Keywords:** tissue spreading, cellular mechanics, lineage diversification

## Abstract

Tissue spreading represents a key morphogenetic feature of embryonic development and regenerative medicine. However, how molecular signaling orchestrates the spreading dynamics and cell fate commitment of multicellular tissue remains poorly understood. Here, it is demonstrated that the dynamic counterbalance between RAC1–YAP and OB‐cadherin plays a key role in coordinating heterogeneous spreading dynamics with distinct cell fate patterning during collective spreading. The spatiotemporal evolution of individual stem cells in spheroids during collective spreading is mapped. Time‐lapse cell migratory trajectory analysis combined with in situ cellular biomechanics detection reveal heterogeneous patterns of collective spreading characteristics, where the cells at the periphery are faster, stiffer, and directional compared to those in the center of the spheroid. Single‐cell sequencing shows that the divergent spreading result in distinct cell fate patterning, where differentiation, proliferation, and metabolism are enhanced in peripheral cells. Molecular analysis demonstrates that the increased expression of RAC1–YAP rather than OB‐cadherin facilitated cell spreading and induced differentiation, and vice versa. The in vivo wound healing experiment confirms the functional role of RAC1–YAP signaling in tissue spreading. These findings shed light on the mechanism of tissue morphogenesis in the progression of development and provide a practical strategy for desirable regenerative therapies.

## Graphical abstract

The collective spreading dynamics and spatiotemporal evolution of stem cell from 3D to 2D is reported. The role of the dynamic counterbalance between RAC1‐YAP and OB‐cadherin in coordinating tissue spreading and stem cell fate patterning is discovered. These findings provide a fundamental basis for advancing deep insight into regenerative medicine.

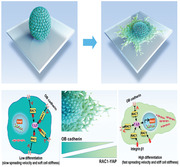

Tissue spreading over a substratum is a fundamental morphogenetic phenomenon in numerous physiological and pathological processes.^[^ ^1^ ^]^ Examples include the development of the embryo, which requires the orchestrated movement of the blastoderm over the yolk sac to shape the body plan of multicellular creatures.^[^ ^1b,c^ ^]^ It is also present in tissue engineering, where stem cell spheroids are introduced to spread in vivo for repairing defects^[^ ^2^ ^]^ or for expanding within an extracellular matrix (ECM) scaffold for recapitulating organ embryogenesis.^[^ ^3^ ^]^ A lack of coordination between the spreading and fate commitment of collective stem cells leads to malformation and dysfunction of the final tissue.^[^ ^4^ ^]^ Understanding the mechanism of how tissue‐scale morphogenesis influences stem cell behavior is critical for improving the therapeutic efficacy for diseases and for advancing fundamental insight into regenerative medicine.

In tissue spreading, the precise control of both the physical and biological characteristics of individual stem cells in space and time enables tissue‐scale morphogenesis and appropriate cell fate patterning. Cell mechanical properties and migratory behaviors are influenced by cell–cell and cell–substrate interactions via external forces, while intracellular protein expression and cell differentiation are regulated by mechanotransduction. Classic physical studies applied physical principles to direct cancer epithelial cell spreading,^[^ ^5^ ^]^ with the proposed mechanism that cells spread collectively like the wetting of a viscous liquid drop, or that the leader–follower cell organization leads to the spreading frontier.^[^ ^6^ ^]^ These studies highlight the mechanical forces and cellular physical properties in directing tissue spreading. However, the biological cell fate commitment of spreading cells and the intracellular molecular mechanism are both lacking. Moreover, despite 2D monolayered cell models demonstrating that several genes are critical for leader or precursor cell organization, such as that for *β*‐actin, Erk1/2, RhoA, and Notch1/Dll4, 3D tissue spreading involves more sophisticated cell behaviors and cell–cell mechanical interactions.^[^ ^7^ ^]^ Hence, how cellular physical characteristics coordinate cell fate commitment in 3D tissue‐scale morphogenesis remains undetermined.

In the present study, we investigated mechanotransduction in coordinating the dynamic spreading and fate commitment of mesenchymal stem cells (MSC) from 3D spheroids to 2D monolayers. Time‐lapse microscopy combined with in situ atomic force microscopy (AFM) were used to track the evolution of collective spreading and to probe the physical properties of MSC in multicellular spheroids. Single‐cell sequencing revealed that cell fate patterning was tightly associated with the cell's position within the spreading spheroid. The underlying cellular signaling events during collective spreading were investigated using molecular analysis and an in vivo wound healing assay. We demonstrate that the counterbalance of RAC1–YAP/OB‐cadherin cascades influence cell fate patterning along with collective spreading. This work sheds light on the dynamics of tissue spreading in the progression of development and in regenerative organization.

Cell spheroids hold great promise for recapitulating the physiological environment of tissue spreading.^[^ ^8^ ^]^ Previous studies on tissue‐scale morphogenesis were mainly derived from epithelial or tumor cells without the differential function. Here, we used multicellular MSC spheroids^[^ ^9^ ^]^ to model embryonic tissue and study their collective spreading dynamics and cell fate commitments. Several studies that have used this model have demonstrated that tissue spreading onto substrates is akin to viscoelastic droplets, following the pioneering work on the liquid‐like behavior of embryonic tissue.^[^ ^1^, ^8^, ^9^ ^]^ In our study, we found that the spreading behavior of spheroids was consistent when the size varied (Figure S1, Supporting Information). Therefore, we developed cell spheroids with 6000 MSCs according to Steinberg's study (**Figure** **1**A).^[^ ^10^ ^]^ The live/dead assay showed that cells in the spheroids with a diameter of 150 µm after 6 h had better biological activity (Figure S2, Supporting Information).^[^ ^2^ ^]^ We seeded the spheroids onto a stiff matrix with 1 MPa elastic modulus to mimic the mechanical microenvironment of bone tissue in vivo.^[^ ^11^ ^]^ The difference between the “center” and the “periphery” was established according to the spreading status of the MSCs in the spheroids. The multi‐layered MSCs in the central zone of the spheroid configuration were defined as the “center”, while the single‐layered MSCs in the peripheral region of the spheroid configuration were defined as the “periphery”. We used two groups of MSCs to aggregate the spheroids to characterize whether cells were patterned to the center or periphery, defined by their prior properties during single cell aggregation. One group was cultured in MSCM (Mesenchymal stem cell media) media while the other one was cultured in the osteogenic inductive media. After 7 days’ culture, the same numbers of these two types of MSCs, dyed with Dil (1,1′‐dioctadecyl‐3,3,3',3'‐tetramethylindocarbocyanine perchlorate) and DiO (3,3′‐dioctadecyloxacarbocyanine perchlorate), respectively, were used for aggregation into spheroids. The immunofluorescence staining showed that the two cell types were distributed almost homogeneously both in the initial spheroid configuration and in the spread‐out spheroid configuration (Figure S3A, Supporting Information). This indicates that the cells are not patterned to location defined by their prior properties. Furthermore, we used 50% MSCs and 50% endothelial cell (ECs) to repeat the above procedure. The same results were achieved in that the MSCs and ECs were distributed almost homogeneously in the spheroid before and after spread‐out (Figure S3B, Supporting Information). Our findings suggest that the diverse properties of the initial population do not affect the way the cells form a spheroid and how these cells are distributed within the spheroid during initial aggregation and hence patterning.

**Figure 1:**
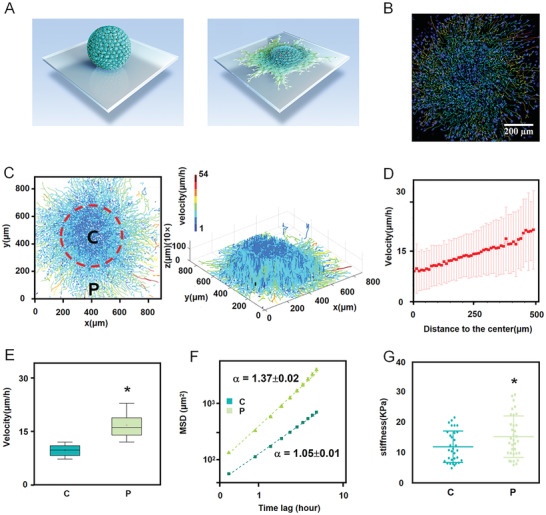
Central and peripheral subpopulations in stem cell spheroid show distinct collective spreading dynamics and cellular biomechanical properties. A) Scheme of MSC spheroid spreading. B) Representative immunofluorescent images for MSC spheroid spreading. C) A heatmap of each single cell's movement trail and speed. D) Quantification of cell spreading velocities showing that cellular velocity increased along with the distance to the spheroid center. The error bar represents the standard deviation. E,F Velocities and MSD curves of cell movements indicating that peripheral cells were faster and in directional motion (*α* = 1.37), whereas central cells were in randomly diffusive motion (*α* = 1.05). The time lag is from 0.5 to 5 h. G) Quantification of MSC stiffness showing that the peripheral cells were stiffer than the central cells, as measured by AFM. Data are the means ± SEM (E, F, G). *P < 0.05; two‐tailed unpaired Student's *t*‐test (E,G).

To study the cell migrations, individual cells were tracked in collective spreading over 13.5 h using 3D time‐lapse confocal imaging (Movie S1, Supporting Information). Cell tracks were constructed by recording cell nuclei every 30 min (Figure 1B), and the velocities of individual cells in the MSC spheroids were calculated in 3D (Figure 1C,D).We found that peripheral cells migrated faster than those in the center of the spheroid (Figure 1E). The mean square displacement (MSD) of trajectories was calculated according to the equation MSD(*τ*) = <(*r*(*t*+*τ*) − *r*(*t*))^2^>, where *τ* is the time lag, and then fitted by the power law MSD(*τ*) = *A*α*τ^*α*^*. The exponent *α* indicates the nonlinear relationship between MSD and time, containing the information of cell motion modes: *α* around 1 indicates randomly diffusive motion; *α* > 1 indicates the directed motion.^[^ ^12^ ^]^ It reveals that peripheral cells migrated in a more directed fashion (*α* = 1.37), whereas central cells were in randomly diffusive motion (*α* = 1.05) (Figure 1F). Note that these trends of the difference in cell dynamics are quite stable, as we obtained similar results by dividing the spreading spheroid into for zones (Figure S4A,B, Supporting Information) . With the AFM mounted on an optical microscope, we used tapping‐mode AFM to detect the stiffness of interested single cells, and found that cell stiffness gradually increased from the center to the periphery of the spheroid (Figure 1G). Moreover, the stiffness of the cells in the periphery of the spheroids was significantly higher than that of cells still in the spheroid configuration (Figure S5, Supporting Information). This phenomenon is because that the proportion of cell–cell interaction in the peripheral cells in initial spheroid configuration is higher than in the spreading cells from spheroid, which inhibit the cellular structure organization and decrease the cell stiffness. Meanwhile, the stiffness of the cells in the periphery of spheroids is comparable to that of the cells cultured on 2D substrates (Figure S5, Supporting Information). This could be ascribed to the dominant role of the cell–substrate interaction in these two situations, which facilitates cellular structure organization and increases cell stiffness. Here, we reveal heterogeneous patterns of cellular physical characteristics, where the stem cells at the periphery are faster and stiffer than those in the center of the spheroid. This evolution from a homogeneous population to heterogeneous one, with divergent dynamic and biomechanical characteristics, is critical for the genetic programming of stem cells, which is observed in the progression of development.^[^ ^4^, ^13^ ^]^ The distribution of cellular biomechanics in our tissue spreading model is opposite to that in the cancer invasion model, where the peripheral cells are softer,^[^ ^4^, ^14^ ^]^ which could be ascribed to the distinct extracellular mechanical environment between the soft ECM in carcinoma and the stiff substrate in bone tissue.

Stem cell spheroids promote significant defect regeneration as compared to cell monolayers.^[^ ^3^, ^15^ ^]^ However, in‐depth spatiotemporal analysis of cell fate commitment in spheroids remains challenging. To investigate stem cell differentiation in the MSC spheroids during spreading, single‐cell sequencing was performed to determine the cell fate commitment in the central and peripheral regions, which were extracted by microdissection after 24 h spreading. Hierarchical gene clustering and pathway analysis showed that there was no significant upregulation of the neurogenic, adipogenic or myogenic transcripts in the central MSCs compared to those in the periphery (Figures S6 and S7, Supporting Information). These results suggest that the stem cell phenotype might be the main population of MSCs in the central spheroids. This view was verified by the almost homogeneous staining of the stem cell marker OCT4 in the central spheroids (Figure S8, Supporting Information). On the contrary, a panel of osteogenic transcripts and cascades (WNT, TGF*β*) (**Figure** **2**A, clusters 5, 6, 7) were activated in peripheral cells than in central cells (Figure 2B), which we found remarkable. This phenomenon was corroborated by the protein secretion of BMP2 (Figure 2E) and the gene expression of BMP2 and COL1A (Figure 2F). Additionally, the immunofluorescence staining after 1‐day and 2‐day culture also showed that the osteogenic‐specific markers RUNX2 and SP7 were significantly upregulated in the peripheral MSCs compared to the central MSCs (Figure S8, Supporting Information). These findings demonstrate that the peripheral MSCs are primed to osteogenic lineage, whereas the central MSCs remain in the stem cell phenotype.

**Figure 2:**
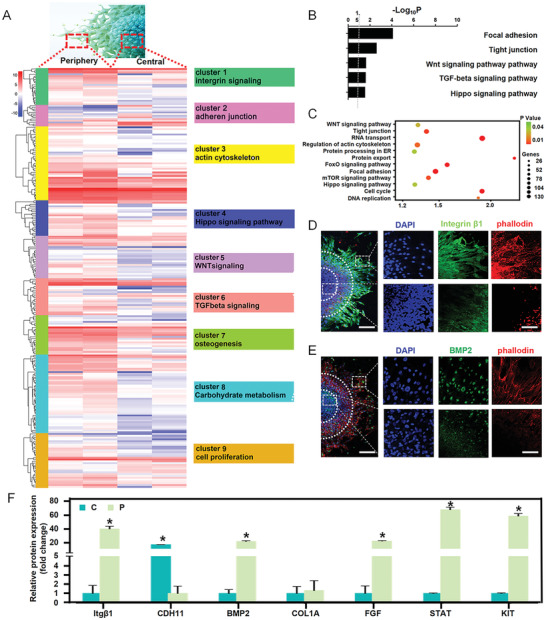
The regional divergent collective spreading resulted in enhanced differentiation, proliferation, and metabolism in peripheral cells rather than in central cells in vitro. A) Hierarchical gene clustering of single‐cell sequencing showing differential peaks [log2(normalized reads in peaks)] between the central and peripheral stem cells. k‐means clusters are indicated by colored bars on the left. Enriched GO terms for each cluster are shown on the right. B,C) GO enriched pathways for upregulated genes in the periphery. D) Immunofluorescence staining showing the upregulation of BMP2 in the periphery. Scale bars, 100 µm in wide‐fields; 25 µm in insets. E) Immunofluorescence staining showing upregulation of integrin *β*1 and cytoskeletal organization in the periphery. Scale bars, 100 µm in wide‐fields; 25 µm in insets. F) RT‐qPCR quantification revealing the upregulation of the marker integrin (Itg*β*1), osteogenic markers (COL1A and BMP2), and cell proliferation markers (FGF, STAT, and KIT) in the periphery, and the upregulation of the adherens junction marker (CDH11) in the center. Data are the means ± SEM. *P < 0.05, one‐way ANOVA (F).

To determine the possible mechanism behind this regionally diverse differentiation during collective spreading from 3D to 2D, we performed in‐depth analysis of the single‐cell sequencing results. Hierarchical gene clustering showed that integrin (ITG) and focal adhesion signaling were upregulated in the periphery rather than in the center (Figure 2A, clusters 1, 2), which indicates that the outside–in mechanical sensing^[^ ^16^ ^]^ is enhanced in peripheral cells due to extensive cell–matrix interaction. Besides, pathway analysis showed that actin organization and Hippo signaling were upregulated in the periphery rather than in the center (Figure 2A, clusters 3,4), suggesting that intracellular mechanical transduction is activated in the peripheral cells. Immunofluorescence staining and qRT‐PCR analysis confirmed these findings (Figure 2D,F). These results demonstrate that the differentiation induced in peripheral cells rather than in central cells can be ascribed to the significant activation of mechanotransduction cascades. Concurrently, gene clustering also revealed that a panel of cell metabolism and proliferation transcripts (Figure 2A, clusters 8, 9) and related signaling pathways (Figure 2C and Figure S9A,B: Supporting Information) were upregulated in the peripheral cells as compared to the central cells. In the EdU (ethynyl deoxyuridine) assay, the peripheral cells had more EdU staining than the central cells (Figure S9C: Supporting Information). The quantified ratio of proliferative cell fluorescence intensity to total cell nuclear fluorescence intensity showed a greatly higher proliferation rate of 33.3% in peripheral cells compared to the 20.9% of the central cells (Figure S9D,E: Supporting Information). Moreover, qRT‐PCR indicated that the proliferation‐related biomarkers fibroblast growth factor (FGF), signal transducer activator of transcription 1 (STAT), and receptor tyrosine kinase (KIT) were all upregulated in peripheral cells rather than in central cells (Figure 2F). The results indicate that there is significant variation of molecule signaling in differentiation, proliferation, and metabolism during collective spreading of the MSC spheroid. Together, the results from single‐cell sequencing, protein secretion, and gene expression indicate that the diversified pattern of cell fate commitment presents in spreading stem cell spheroids, and coincides with the heterogeneous distribution of the cellular physical characteristics of cell motility and stiffness shown in Figure 1. The significantly enhanced differentiation, proliferation, and metabolism enable peripheral cells to migrate faster and behave more stiffly than cells in the center of the spheroid. It is also suggested that the heterogeneous distributions of physical and biological behaviors in stem cell spheroids may simulate the natural processes of tissue regeneration and benefit defect repair.^[^ ^17^ ^]^

Having established the heterogeneous patterns of physical spreading dynamics and the distinct manner of biological cell fate commitment during spheroid spreading, we further investigated the molecular mechanisms that coordinate these two aspects. As the single‐cell sequencing suggested significant activation of mechanotransduction in peripheral cells rather than in central cells, we focused on key molecular switches in converting and amplifying external force into intracellular signaling. Both gene clustering in single‐cell sequencing (**Figure** **3**A) and gene expression by qRT‐PCR (Figure 3I) showed that the expression of the Rho GTPase family (including RAC1, RHOA, ROCK1) and YAP were upregulated in peripheral cells rather than in central cells. Immunofluorescence staining confirmed these results. Interestingly, we found that RAC1, YAP, F‐actin, and integrin expression gradually increased from the center to the periphery, whereas OB‐cadherin expression gradually decreased (Figure 3C,E,F). This regional divergence is consistent with the results of the scatter plot (Figure 3B). Moreover, the distribution of these molecular signals is similar to the heterogeneous patterns of cellular physical and biological behaviors the spheroids (Figure S4, Supporting Information). The above results suggest that these multi‐signals might be the mechanism underlying mechanism.

**Figure 3:**
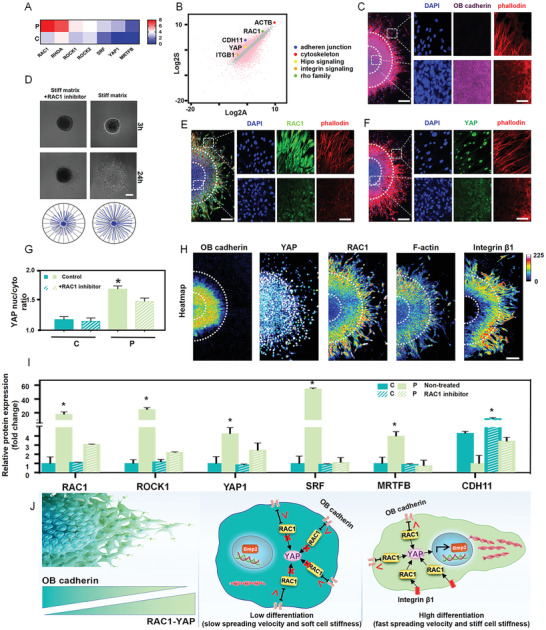
The dynamic counterbalance between RAC1–YAP and OB‐cadherin coordinates collective spreading and cell fate patterning. A) Hierarchical gene clustering and B) scatter plot demonstrating that small GTPase transcripts were upregulated in the periphery. C) Immunofluorescence staining showing that OB‐cadherin was upregulated in the center after 24 h spheroid spreading. Scale bars, 100 µm in wide‐fields, 25 µm in insets. D) Spreading trail of spheroids indicating that treatment with the RAC1 inhibitor reduced spheroid movement. E) Immunofluorescence staining showing that RAC1 was upregulated in the periphery after 24 h spheroid spreading. Scale bars, 100 µm in wide‐fields, 25 µm in insets. Immunofluorescence staining of YAP nuclear translocation F) and quantification of YAP nuclear‐to‐cytoplasmic ratios G) showing enhanced YAP expression and nuclear translocation in the periphery, while the inhibition of RAC1 decreased it significantly. Scale bars, 100 µm in wide‐fields; 25 µm in insets. H) Protein expression immunofluorescence heatmap revealing the gradient molecular expression pattern. Scale bar, 100 µm. I) RT‐qPCR quantification revealing the downregulation of small GTPase transcript markers (RAC1, ROCK1, SRF, MRTFB) and YAP1 after RAC1 inhibition. J) Schematic presentation of how the dynamic counterbalance between RAC1–YAP and OB‐cadherin coordinates collective spreading and cell fate patterning. Data are the means ± SEM. *P < 0.05, one‐way ANOVA (C, G, I).

RAC1 is the key molecular switch in converting and amplifying external force into cellular biology. OB‐cadherin is a typical transmembrane protein that forms adherent junctions between cells. It has been established that OB‐cadherin is closely relevant to the developmental biology events of mesenchymal condensation, which would be a close paradigm for MSCs aggregation and disaggregation.^[^ ^18^ ^]^ The opposite distributions between RAC1 and OB‐cadherin suggest their major but contrary roles in determining cell fates and behaviors during MSC spheroid spreading. To prove our hypothesis, we performed gain‐ and loss‐of‐function experiments. Distinct dynamics were observed, where the central area with high OB‐cadherin expression could be enlarged or reduced by inhibiting and activating RAC1, respectively (Figures S10D and S11A, Supporting Information). This finding was corroborated by the result showing that the peripheral area with high RAC1 expression could be enlarged by the inhibition of OB‐cadherin (Figure S12A,C, Supporting Information). This suggests a counterbalancing relationship between OB‐cadherin and RAC1.^[^ ^18b^ ^]^ It should be noted that N‐cadherin can inhibit RAC1 expression in separate cells to slow cell motility.^[^ ^19^ ^]^ However, we found no obvious difference in N‐cadherin between the peripheral and central MSC spheroid during spreading in the present study. Our results therefore suggest that OB‐cadherin is the major sensor of cell interaction in MSC rather than N‐cadherin.

Moreover, we noted that YAP showed a similar expression pattern to RAC1 (Figure 3F,H). YAP is a key molecular switch in manipulating cell motility and mediating stem cell differentiation.^[^ ^20^ ^]^ RAC1 can manipulate nuclear translocation of YAP by changing nuclear pore size through cytoskeletal organization, or it can directly activate YAP through protein binding.^[^ ^17^, ^21^ ^]^ The inhibition of RAC1 decreased the nuclear translocation of YAP (Figure 3G and Figure S10A: Supporting Information) and differentiation marker expression (Figure S10C, Supporting Information), while RAC1 activation facilitated it (Figure S11B,C, Supporting Information). The results indicate that RAC1–YAP cascade signaling takes place in the diverse cell fate commitment during MSC spheroid spreading.

How does this molecule signaling coordinate the heterogeneity of cellular physical and biological behavior? We propose a possible model: the counterbalance between RAC1–YAP and OB‐cadherin. In the periphery, as cells reach the stiff 2D substrate from a 3D multicellular spheroid, there is greater activation of RAC1 intracellular expression, probably due to the significant upregulation of ITG*β*1 on the membrane by the extensive cell–substrate interaction.^[^ ^21^ ^]^ Downstream, the highly activated RAC1 can promote actin polymerization and also increase intranuclear YAP. Meanwhile, RAC1 suppresses the OB‐cadherin junctions, decreasing cell–cell interactions. As the actin cytoskeleton plays major roles in cellular biomechanics and in cell motility, the peripheral cells, with promoted actin polymerization, exhibit relatively high motility and stiffness. Besides, the more intranuclear YAP in the peripheral cells promote their osteogenic differentiation.^[^ ^22^ ^]^ which can further increase cell motility and stiffness. From the periphery to the center, more cells in the 3D multicellular spheroid lack interaction with the substrate. Therefore, there is less activation of RAC1, decreasing both actin polymerization and the amount of intranuclear YAP. However, more OB‐cadherin junctions are generated, increasing cell–cell interactions. In contrast to the periphery, the central cells show, physically, decreased motility and stiffness, and biologically, less osteogenic differentiation. Together, the dynamic counterbalance between RAC1–YAP and OB‐cadherin coordinates collective spreading and cell fate patterning. The overwhelming expression of RAC1–YAP compared to OB‐cadherin can facilitate cell directional spreading and induce differentiation in the periphery, and vice versa in the center.

The counterbalance between RAC1–YAP and OB‐cadherin was demonstrated by the fact that the inhibition of RAC1 by NCS23766 or small interfering RNA (siRNA) decreased F‐actin organization, cellular stiffness (Figure S5A, Supporting Information), spreading velocity (Figure 3D and Figure S9E: Supporting Information), and RAC1 downstream gene expression (Figure 3I and Figure S13: Supporting Information), while they were facilitated by activated RAC1 (Figure S11D,E: Supporting Information). Moreover, the inhibition of YAP activation by YAP/TAZ inhibitor‐1 not only reduced the expression of differentiation markers (Figure S14A: Supporting Information), but also significantly decreased cell stiffness and spreading velocity (Figure S13B,C, Supporting Information).

To explore the mechanotransduction role of the RAC1–YAP cascades in tissue spreading, we investigated MSC spheroid spreading on a softer substrate (15 kPa) with mechanical properties mimicking that of connective tissue.^[^ ^11^ ^]^ As there was less integrin on the soft substrate, RAC1 and YAP were expressed at very low levels (**Figure** **4**A,C) while the spheroid spread area and velocity were reduced by 60% compared with that on stiff matrix (Figure 4D,E). Here, we rescued RAC1 by activating epidermal tyrosine kinase receptors through an epidermal growth factor (EGF) agonist. Strikingly, both immunofluorescence and qRT‐PCR analysis showed that activating RAC1 significantly increased YAP expression and actin organization (Figure 4A,C,F). Moreover, the velocity of collective spreading was also upregulated (Figure 4D). These results demonstrate that mechanical cell–substrate interaction and RAC1–YAP signaling transduction play critical roles in determining the heterogeneous MSC behaviors in spheroid spreading.

**Figure 4:**
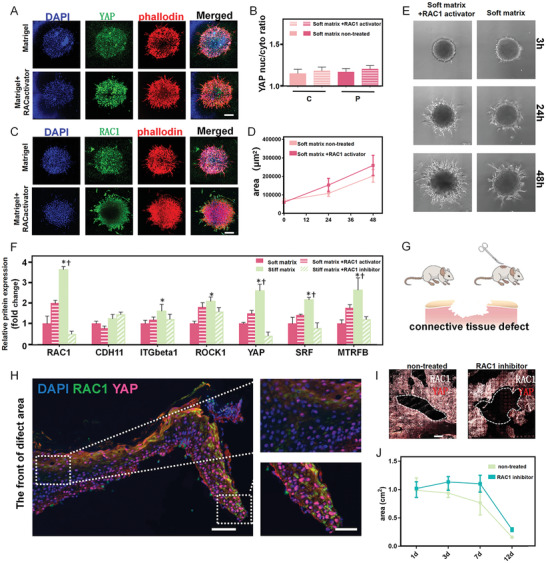
The influence of RAC1‐YAP signaling regulates collective spreading in vitro and wound healing in vivo. A) Immunofluorescence staining showing that activation of RAC1 enhanced YAP expression and actin organization on soft substrates. Scale bar, 100 µm. B) Treatment with RAC1 activator increased YAP nuclear‐to‐cytoplasmic ratios in the periphery and the center. C) Immunofluorescence staining showing that the activation of RAC1 enhanced RAC1 expression and actin organization on soft substrates. Scale bar, 100 µm. D,E) Quantification of spreading area and spreading trail of spheroids indicating that RAC1 activation enhanced cell movement on soft substrates. F) RT‐qPCR quantification revealing the downregulation of small GTPase transcript markers (RAC1, ROCK1, SRF, MRTFB) and YAP on the soft substrate, with significant upregulation after the activation of RAC1. G) Model of connective tissue defect on mouse skin. H) Immunofluorescence staining showing that the expression of RAC1–YAP signaling gradually decreased from the regeneration boundary to the back. Scale bars, 100 µm in wide‐fields; 50 µm in insets. The top view of immunofluorescence staining of the entire defect area I) and quantitative analysis of the mouse skin defect area J) indicate that RAC1 inhibition prohibited healing of the collective tissue defect. Scale bar, 1 mm. Data are the means ± SEM. *P < 0.05 versus corresponding soft matrix group, †*P* < 0.05 versus corresponding stiff matrix+RAC1 inhibitor group, one‐way ANOVA.

We also examined whether similar RAC1–YAP cascades exist in real tissue spreading. We used an in vivo wound healing model of mouse skin defect (Figure 4G). After 7 days, the wound tissues were fixed and imaged under confocal microscopy. We found that RAC1 and YAP were highly expressed in the boundary of the regeneration area and gradually decreased from the leading edge toward the back (Figure 4H), which is consistent with the in vitro results and suggests that RAC1–YAP cascades are critical in real tissue regeneration. We verified this when we found that inhibiting RAC1 significantly slowed the wound healing (Figure 4I,J). In particular, at 12 days, the defect area in the RAC1 inhibition group was 40% larger than that in the control group (Figure 4J). These findings suggest that RAC1–YAP signaling is important for facilitating collective spreading dynamics and fate commitment in tissue regeneration.^[^ ^23^ ^]^

In summary, we report for the first time the collective spreading dynamics and fate patterning evolution of MSC in space and time, from 3D spheroid to 2D monolayer. Our findings reveal the heterogeneous patterns of spreading dynamics and the coordinated distinct manner of cell fate commitment. The cells in the periphery are faster, stiffer, and directional compared to those in the center of the spheroid. Meanwhile, differentiation, proliferation, and metabolism are significantly enhanced in peripheral cells rather than in central cells. Furthermore, we discovered the critical role of the dynamic counterbalance between RAC1–YAP and OB‐cadherin in coordinating tissue spreading and stem cell fate patterning. The overwhelming expression of RAC1–YAP over OB‐cadherin facilitates cell migration and promotes osteogenic differentiation, and vice versa. Moreover, we confirm the functional role of RAC1–YAP signaling in tissue spreading via an in vivo wound healing experiment on mouse skin. These findings on how tissue‐scale morphogenesis influences stem cell behavior will provide a fundamental basis for improving the therapeutic efficacy for diseases and for advancing deep insight into regenerative medicine.

## Experimental Section

##### MSC Culture and Cell Spheroid Construction

Agarose solution (2%) was melted and then slowly added to a MicroTissues 3D Petri Dish (Sigma). Then, the agarose was solidified into the cell culture mold. MSC (ScienceCell) were inoculated in the agarose mold. After 12 h culture in the mold with MSC medium (ScienceCell), the cells were agglomerated into cell spheres.

##### Time‐Lapse Imaging

Live MSC were labeled using Hoechst 33 258 (Solarbio). A confocal microscope (Nikon A1R HD25) equipped with a live cell platform was used to record the dynamic movement of the cell spheroids every 30 min for 12 h. The obtained motion video was analyzed using Imaris software to obtain the velocity, trajectories, and MSD value.

##### Single‐Cell Sequencing

In the absence of RNase, about 20–30 cells in the periphery and center were cut using a microdissection knife (Head Biotechnology). Complementary DNA (cDNA) was synthesized using a SMART‐Seq v4 single cell kit (Takara). A single‐cell transcriptome library was constructed. After the library had passed quality inspection, the Illumina sequencing platform was used for high‐throughput sequencing with a read length of PE150. The sequencing results were analyzed using gene ontology (GO) enrichment analysis and pathway enrichment analysis.

##### Cell Stiffness

Cell stiffness on a stiff matrix was detected using an atomic force microscope (Bruker). PF *θ* nm‐lc‐a‐cal, LC4 probe were selected, and the set parameters as follows: sum = 4.1 V (25.4 °C), calibrate with no touch, *k* = 0.104 n m^−1^, Def.sens. = 23.67 nm V^−1^. Probe parameter settings: Tip half angle = 18°, Poisson's ratio = 0.5, scan size = 500 nm (there was no scanning morphology, so it can be set larger to ensure that the X rotation in the ramp can reach 12°), peak force frequency = 1 kHz, peak force amplitude = 300 nm, engage setting amplitude = 300 nm. Ramp parameters: ramp size = 1.5 µm; ramp rate = 1 Hz; X rotate = 12°; Z closed loop open; relative contact mode; contact range = 6 nm.

##### Immunofluorescence Analysis

The samples were rinsed with phosphate‐buffered saline (PBS) and fixed in 4% paraformaldehyde for 30 min at room temperature. Then, the samples were permeabilized with 0.1% Triton X‐100 (diluted with PBS) for 10 min and blocked with 3% bovine serum albumin (BSA; diluted with PBS) for 1 h at room temperature. The permeabilization solution was removed and the samples were rinsed with PBS for 5 min at room temperature. The 3% BSA was used for reducing nonspecific staining. The samples were incubated with the following primary antibodies in 5 wt% BSA in PBS overnight at 4 °C: polyclonal rabbit anti‐ITG*β*1 (1:100; abcam), polyclonal rabbit anti‐BMP2 (1:200; abcam), monoclonal rabbit anti‐RAC1 (1:250; abcam), and polyclonal rabbit anti‐YAP1 (1:500; abcam). After thorough rinsing to remove excess antibody, the cells were incubated with the following secondary antibodies for 1 h in the dark: donkey anti‐rabbit IgG H&L Alexa Fluor 594 (1:500; abcam) and goat anti‐rabbit IgG H&L Alexa Fluor 488 preadsorbed (1:500; abcam). Phalloidin (Sigma) was used for cytoskeletal staining. Nuclei were stained using 4’,6‐diamidino‐2‐phenylindole (DAPI; Sigma). Images were captured using a confocal laser scanning microscope (Leica).

##### RT‐qPCR

RNA was extracted using Quick‐RNA MiniPrep Plus (Zymo Research). Reverse transcription was performed using a PCR thermal cycler (Takara). Optical 96‐well reaction plates (Thermo Fisher Scientific) and optical adhesive films (Thermo Fisher Scientific) were used for the PCR. The PCR mixture loaded in each well had a final volume of 20 µL, and included 8 µL FastStart Universal SYBR Green Master Mix (Rox), 10 µL RNase‐free water, 1 µL template cDNA, and 1 µL primer. PCR amplification was conducted with the following cycling parameters: 15 min at 95 °C (heat activation step), followed by 40 cycles of 15 s at 95 °C and 1 h at 60 °C. Data were analyzed using QuantStudio Design and Analysis desktop software (Thermo Fisher Scientific). Differences in gene expression levels among different groups were statistically analyzed. **Table** **1** shows the primer sequences. Glyceraldehyde‐3‐phosphate dehydrogenase (GAPDH) served as the internal control.

**Table 1:** Primers used for quantitative real‐time PCR analysis

Target gene	Forward sequence (5′‐3′)	Reward sequence (5′‐3′)
Integrin *β* 1	GTGAAGCCAGCAACGGACAG	TTTGCCCTTGAAACTTCGGA
CDH11	TATCATCAGAACAGCCCTACCCA	TCACTCTTCCTACTTCCTCCCC
BMP2	AACAATGGCA TGATTAGTGG	CAGACGGGAG TTTCTCCTCGGACGT
FGF	ACAGGAGCGACCAGCACATTCA	CATTCCTCATTTGGTGTCTGTGAGC
Colla1	AAGACGAAGACATCCCACCAATC	CAGATCACGTCATCGCACAACA
MTRFB	GGTGAAGCAAAGCCATCCC	AGGCGGGCGTAGTTAGAGTC
RAC1	TGATGCAGGCCATCAAGTGT	AGAACACATCTGTTTGCGGA
KIT	TGGGCGACGAGATTAGGCT	AAGGAGCGGTCAACAAGGAA
STAT1	TGGAGTGGAAGCGGAGACAG	GTGATAGGGTCATGTTCGTAGGTGTA
YAP	GTCTTCCTTTGAGATCCCTGA	CTGCCATGTTGTTGTCTGAT
GAPDH	CCTGGGCTACACTGAGGACC	CATACCAGGAAATGAGCTTCAC

##### Skin Wound Healing Model

Healthy 5‐week‐old C57 mice (Charles River Laboratories) were used in this model. A 1% sodium pentobarbital solution was used for anesthesia. A square skin lesion with a diameter of 1 cm was made. Then, the wound was protected using surgical dressing and penicillin injection to prevent infection. The RAC1 inhibitor NSC23766 (2.5 mg kg^−1^, MCE) was given to the RAC1 inhibitor group. On day 3, the drug was given daily until the wound healed. On day 7 after the operation, three mice were killed by excessive anesthesia. The skin of the defect area was fixed in 4% paraformaldehyde for 24 h. RAC1/YAP expression and distribution were observed using immunofluorescence staining. All animal experiments were conducted with the approval of the Animal Care and Use Committee of Peking University (IACUC number: LA2020432).

## Conflict of Interest

The authors declare no conflict of interest.

## Supporting information

Supporting Information

Supplemental Movie 1

## Data Availability

Research data are not shared.
