# Supplementary material for: The Dynamic Counterbalance of RAC1‐YAP/OB‐Cadherin Coordinates Tissue Spreading with Stem Cell Fate Patterning
Source: Adv Sci (Weinh). 2021 Mar 8;8(10):2004000. doi: 10.1002/advs.202004000 (PMC8132063; doi:10.1002/advs.202004000)
Supplement: Supplementary file 1 — Supporting Information [file ADVS-8-2004000-s001.pdf]

© 2021 Wiley-VCH GmbH

ADVANCED SCIENCE
Open Access

Supporting Information

for *Adv. Sci.*, DOI: 10.1002/advs.202004000

The dynamic counterbalance of RAC1-YAP/OB-cadherin coordinates tissue spreading with stem cell fate patterning

Shengjie Jiang, Hui Li, Qiang Zeng, Zuohui Xiao, Xuehui Zhang, Mingming Xu, Ying He, Yan Wei and Xuliang Deng**

Supplementary Information

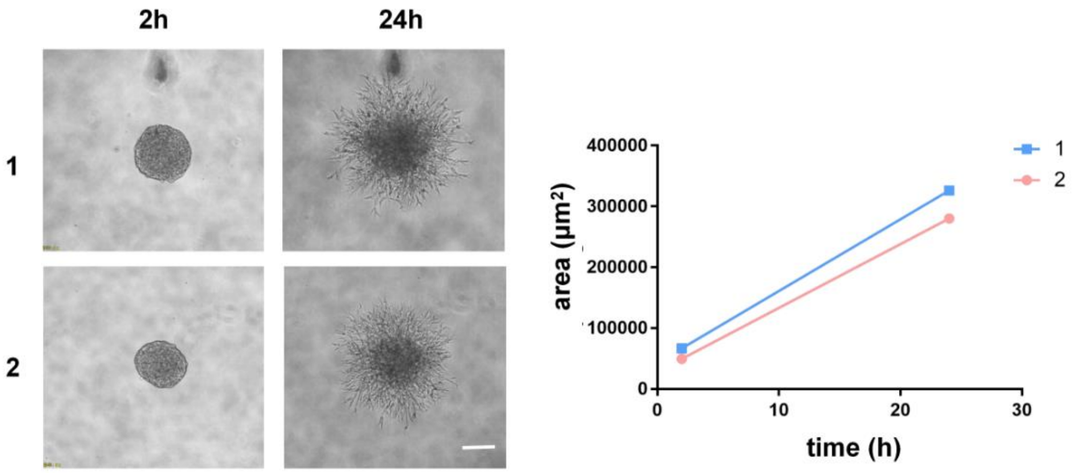

Figure S1. Quantification results of spreading area and spreading trail of different-sized spheroids. The spreading behaviors were qualitatively consistent with each other when the spheroid size was varied. Scale bars, 100 μm.

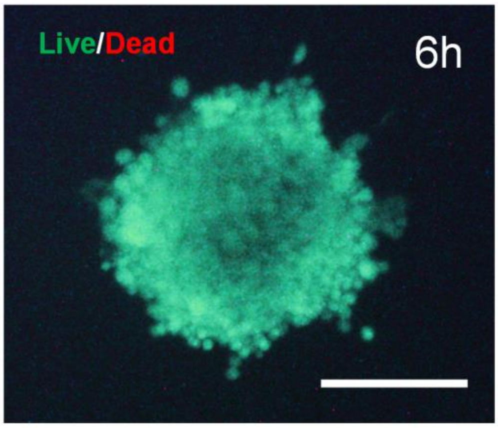

Figure S2. Cell viability determined by live/dead cell staining.

Live/dead assay (calcein-AM) performed after 6-h culture in growth medium indicated the excellent viability of the human MSC (hMSC) spheroids. Scale bars, 100 μm.

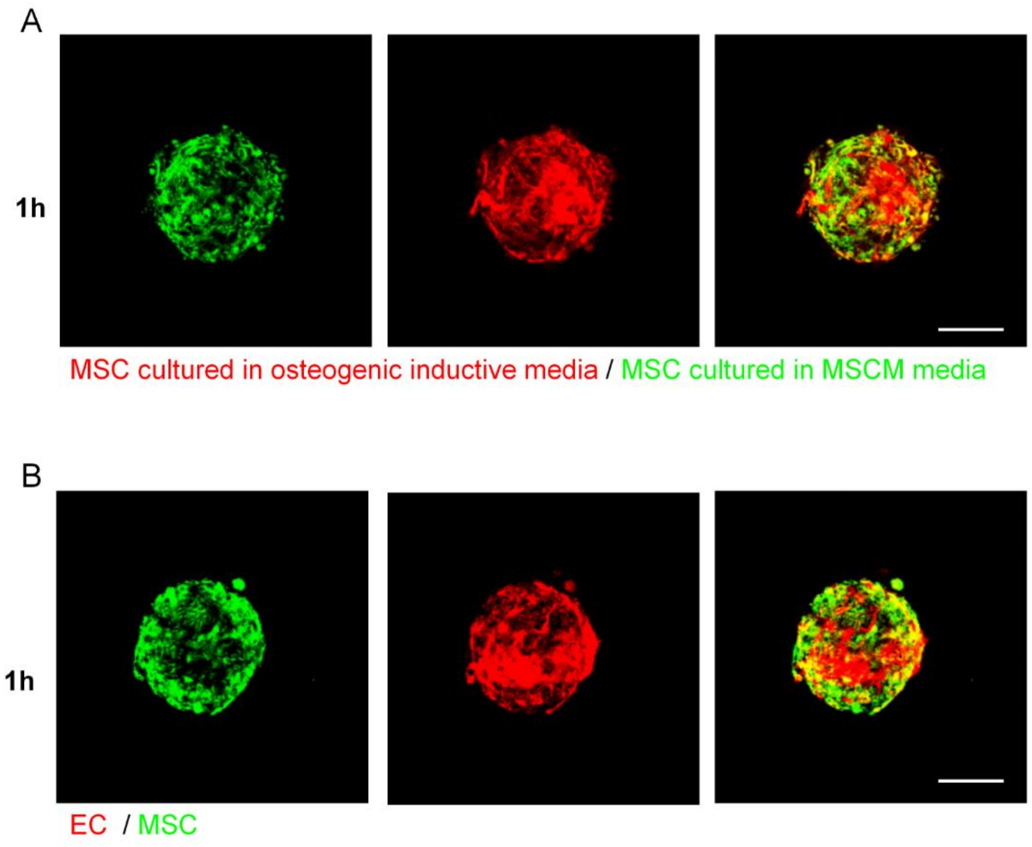

Figure S3. Cell distribution view by Immunofluorescence staining.

(*A*) MSCs cultured in MSC media (green) and MSCs cultured in osteogenic inductive media (red) were distributed almost homogeneously in the spheroid. Scale bars, 100 μm. (*B*) MSCs (green) and ECs (red) were distributed almost homogeneously in the spheroid. Scale bars, 100 μm.

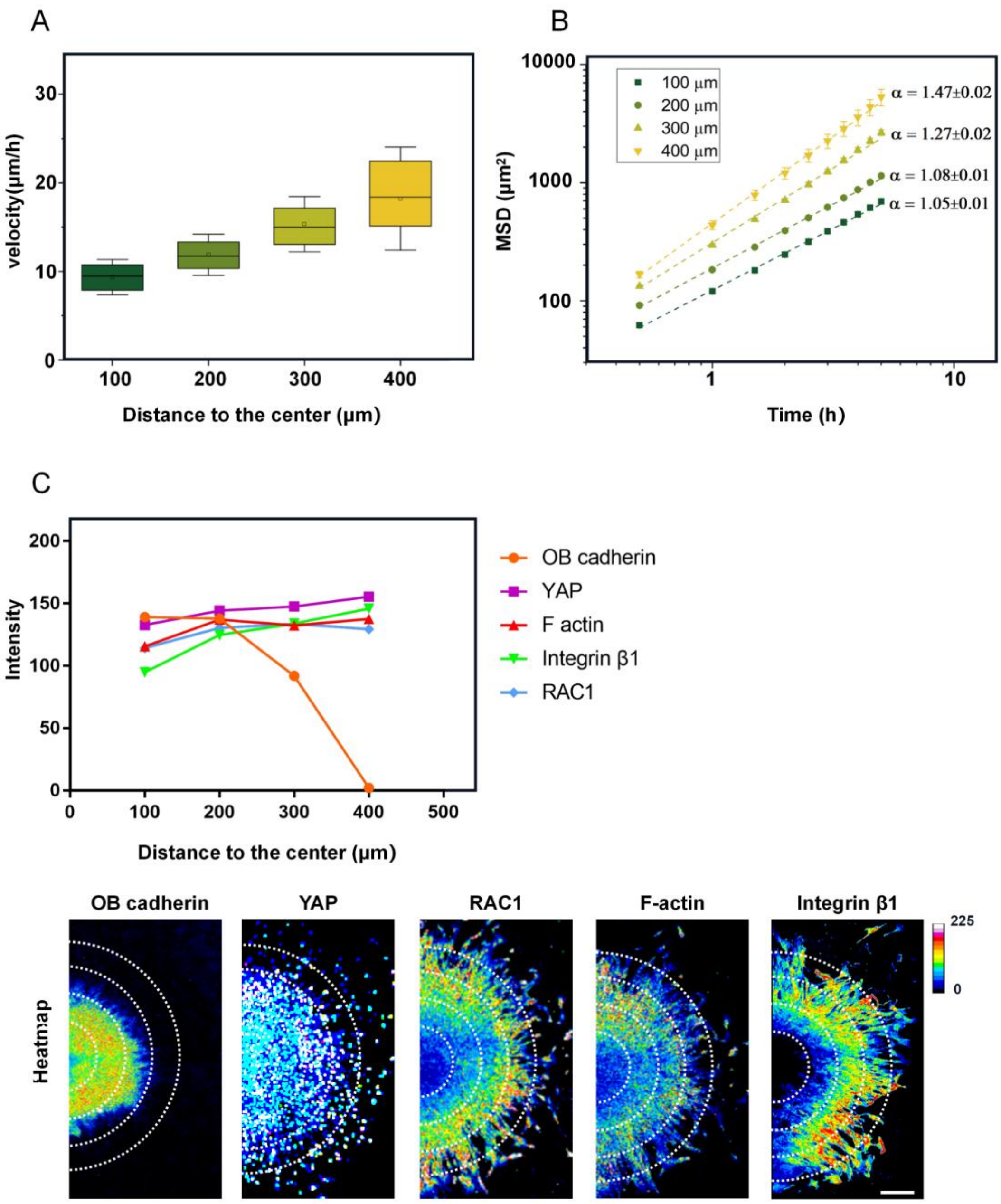

Figure S4. The quantitative analysis curves of the cellular migrations and the proteins expressions in four zones.

The (A)velocity, (B)MSD and the (C)immunofluorescence staining quantification trends were similar. Scale bars, 100 μm.

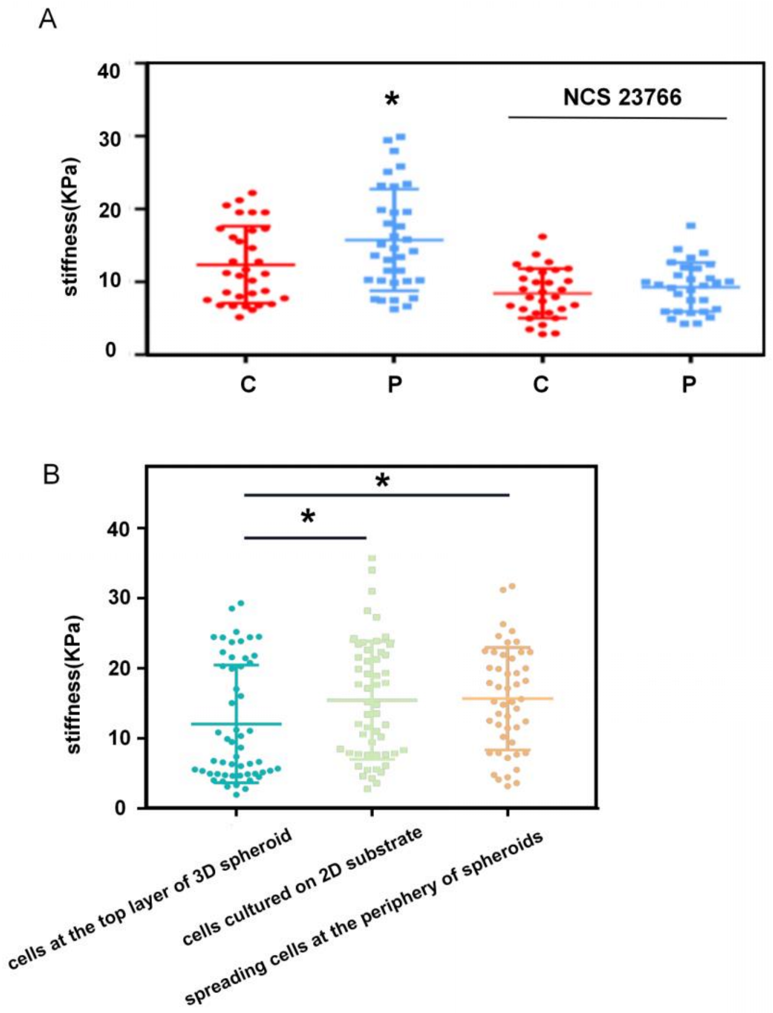

Figure S5. Cellular stiffness detected by AFM.

(A) The inhibition of RAC1 (NCS 23766) decreased cellular stiffnessof the spheroid.; **P* < 0.05 vs. corresponding RAC1 inhibited P group; two-tailed unpaired Student's *t*-test. (B) The stiffness of cells cultured on 2D substrate and The stiffness of spreading cells at the periphery of spheroids were both significantly higher than that of cells at the top layer of 3D spheroid ;**P* < 0.05, two-tailed unpaired Student's *t*-test.

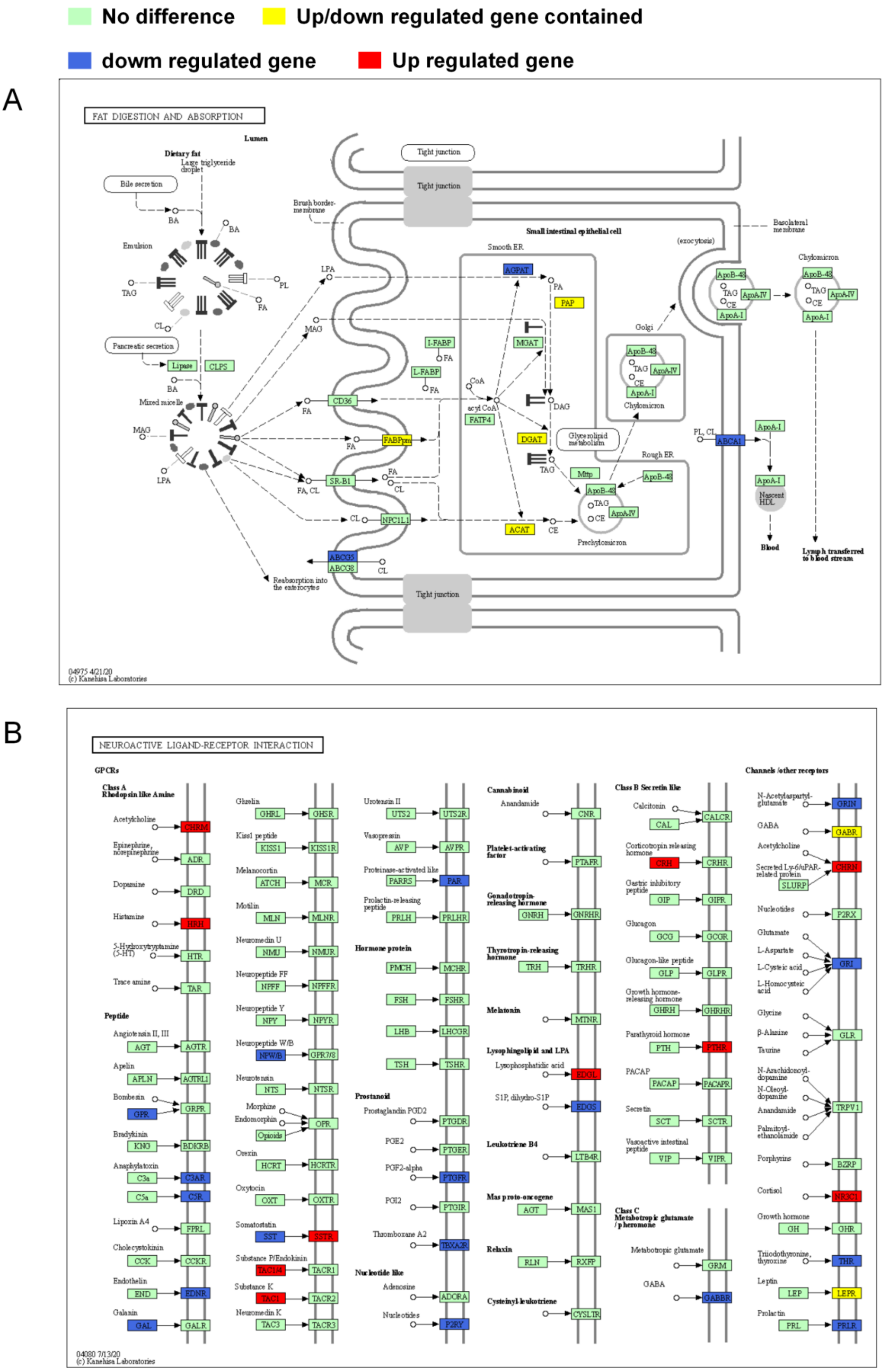

Figure S6. Genome analysis of cell differentiation potential.

(A, B) KEGG cascades analyses showed that there was no significant upregulation of the neurogenic, adipogenic transcripts in central MSCs compared to those in periphery.

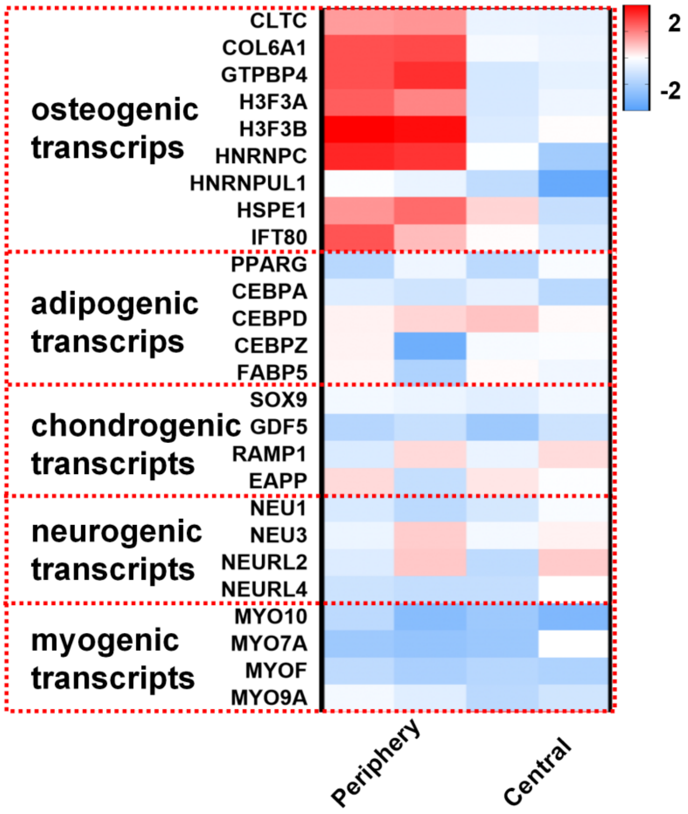

Figure S7. Genome analysis of cell differentiation potential.

Hierarchical clustering analyses showed that there was no significant upregulation of the neurogenic, adipogenic or myogenic transcripts in central MSCs compared to those in periphery.

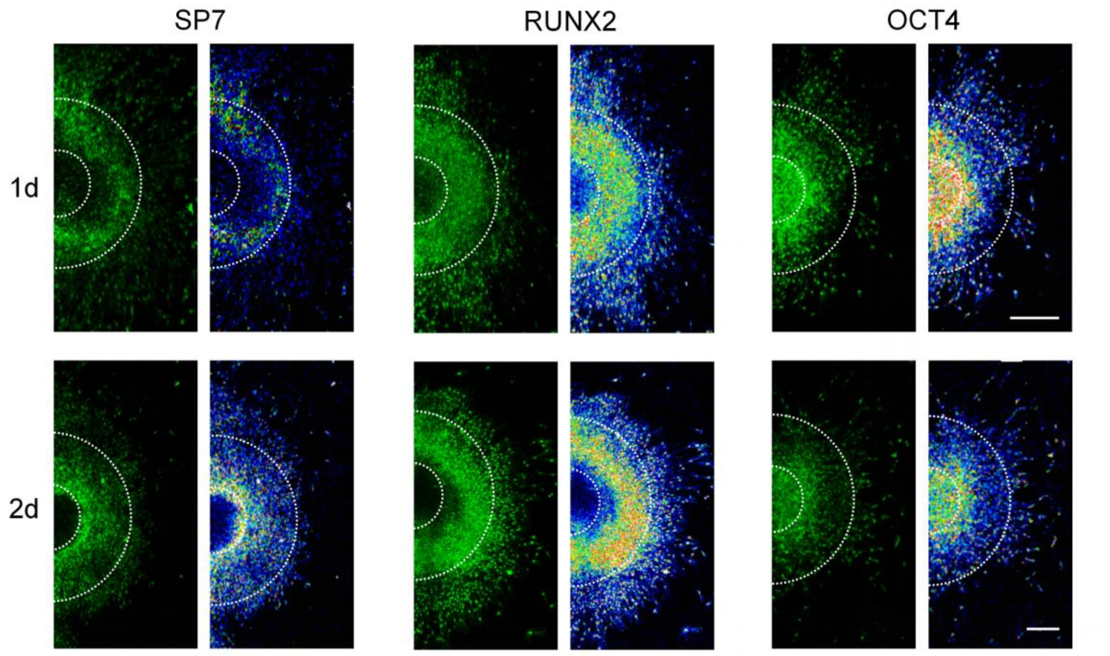

Figure S8. Immunofluorescence staining and heatmap of osteogenic specific markers.

SP7, RUNX2 and OCT4 was upregulated in the periphery after 1 d and 2 d spheroid spreading. Scale bars, 100 μm.

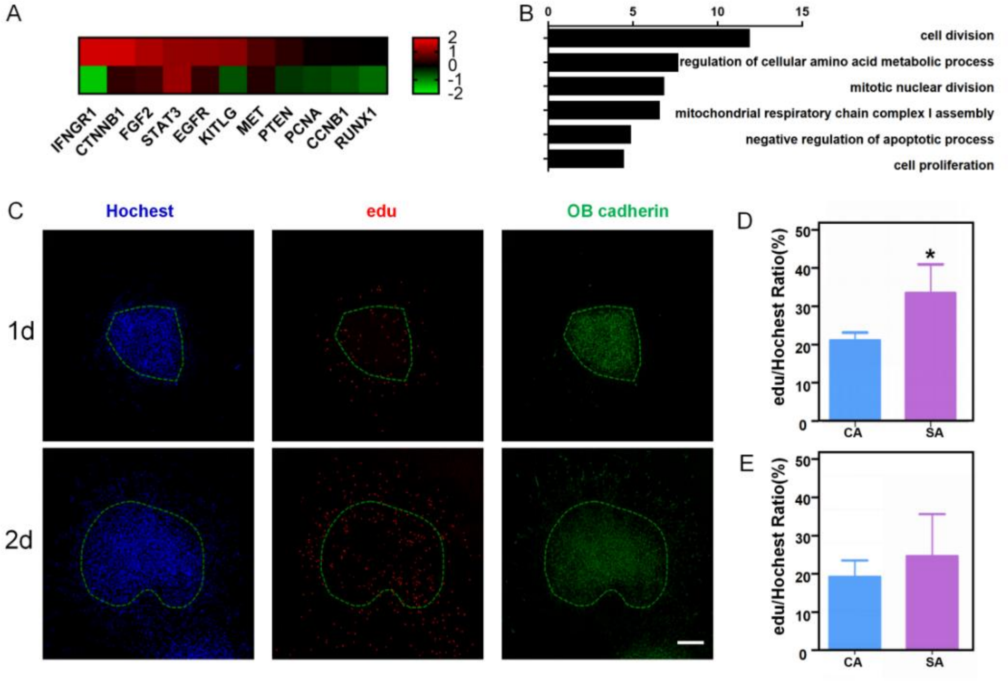

Figure S9. Cell metabolism and proliferation determined by gene clustering and EdU assay.

(*A*, *B*) Gene clustering revealed that a panel of cell metabolism and proliferation-related signal pathway proteins were upregulated in the peripheral cells as compared to the central cells. Scale bars, 100 μm. (*C*–*E*) EdU assay and quantitative analysis of the EdU/Hoechst ratio indicated that the peripheral cells had more EdU staining than the central cells. Cell Counting Kit-8 (CCK-8) assay found no significant difference. *$P < 0.05$, two-tailed unpaired Student's *t*-test.

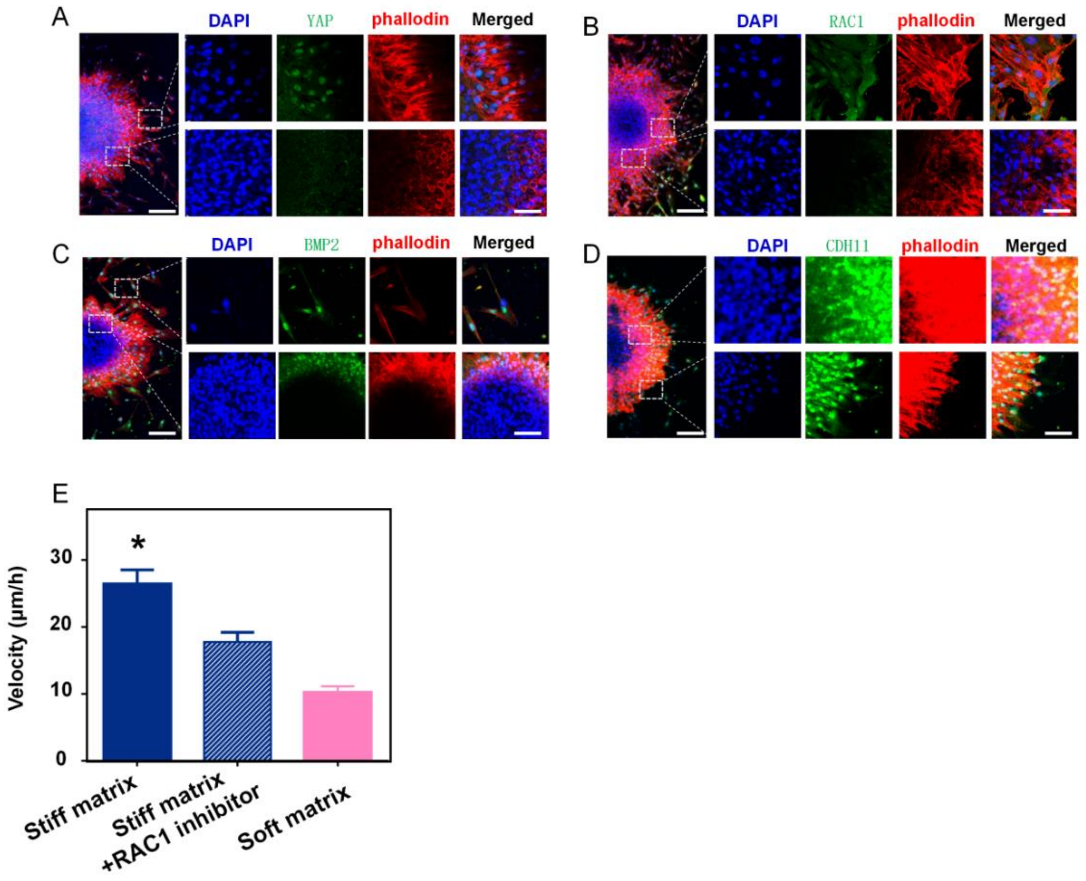

Figure S10. RAC1 loss-of-function by NSC23766 treatment.

(*A*) The inhibition of RAC1 decreased YAP nuclear translocation. Scale bars, 100 µm in wide-fields; 25 µm in insets. (*B*) The inhibition of RAC1 decreased RAC1 in the peripheral cells. Scale bars, 100 µm in wide-fields; 25 µm in insets. (*C*) The inhibition of RAC1 decreased the expression of the differentiation marker BMP2 in the peripheral cells. Scale bars, 100 µm in wide-fields; 25 µm in insets. (*D*) The inhibition of RAC1 could increase OB-cadherin. Scale bars, 100 µm in wide-fields; 25 µm in insets. The inhibition of RAC1 decreased spreading velocity (*E*).**P* < 0.05 vs. corresponding stiff matrix +RAC1 inhibitor group, two-tailed unpaired Student's *t*-test.

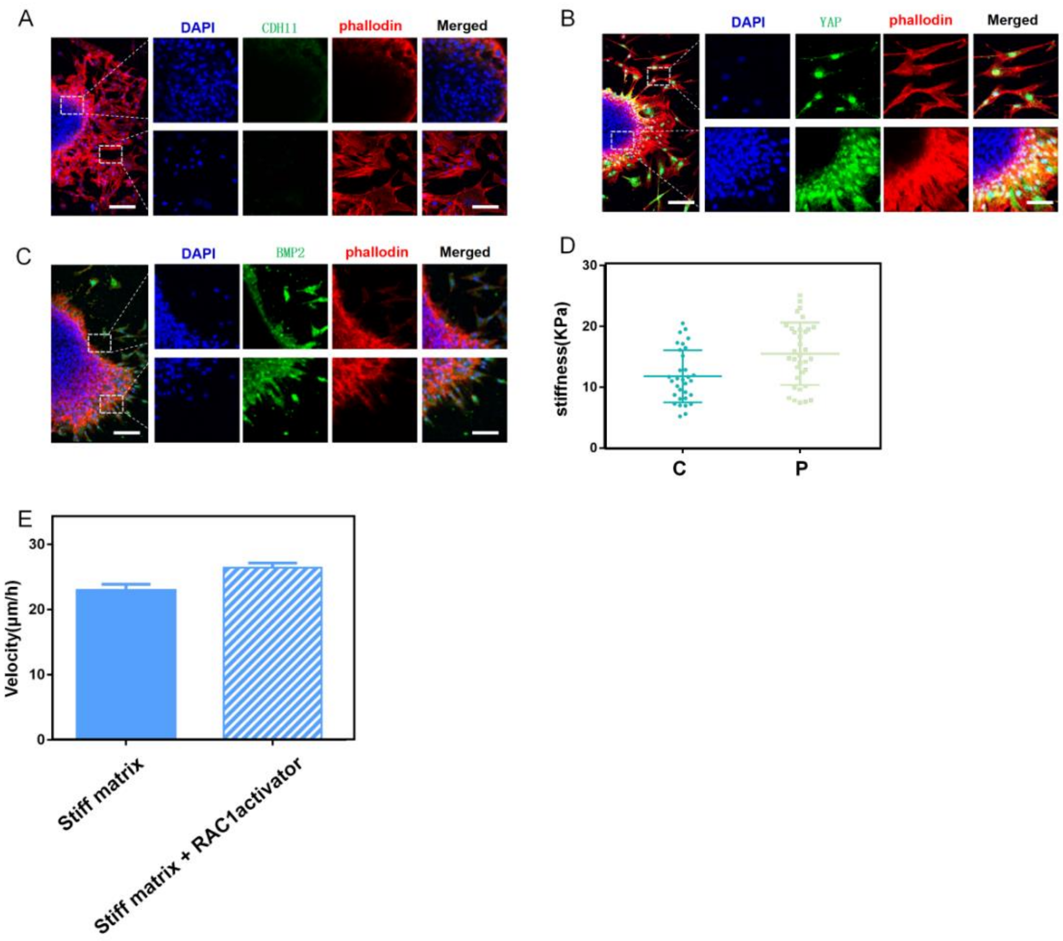

Figure S11. RAC1 gain-of-function by epidermal growth factor treatment.

(*A*) The activation of RAC1 could reduce OB-cadherin. (*B*) The activated RAC1 facilitated YAP nuclear translocationand (C) differentiation marker expression . (*D*)The activation of RAC1 increased cellular stiffnessand (E) spreading velocity. Scale bars, 100 µm in wide-fields; 25 µm in insets.

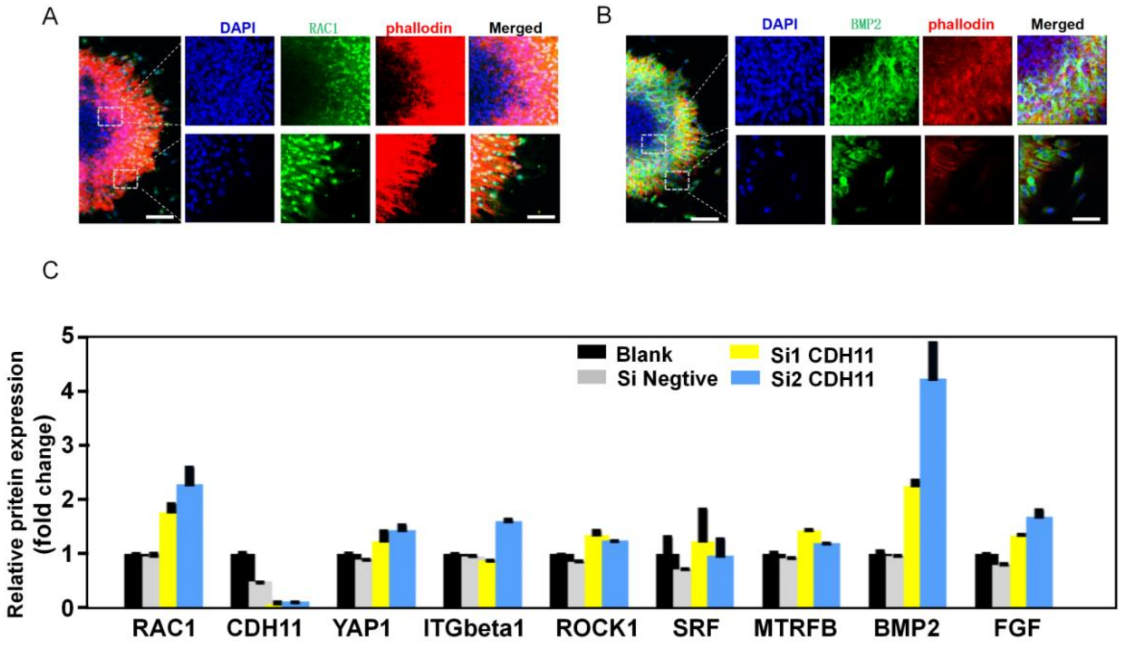

Figure S12. The loss-of-function of OB-cadherin by CDH11 siRNA.

(*A*) siRNA inhibition of *CDH11* enlarged the peripheral area with high RAC1 expression. Scale bars, 100 µm in wide-fields; 25 µm in insets. (*B*) siRNA inhibition of *CDH11* enlarged the peripheral area with high expression of BMP2. Scale bars, 100 µm in wide-fields; 25 µm in insets. (*C*) siRNA inhibition of *CDH11* increased RAC1 downstream gene expression.

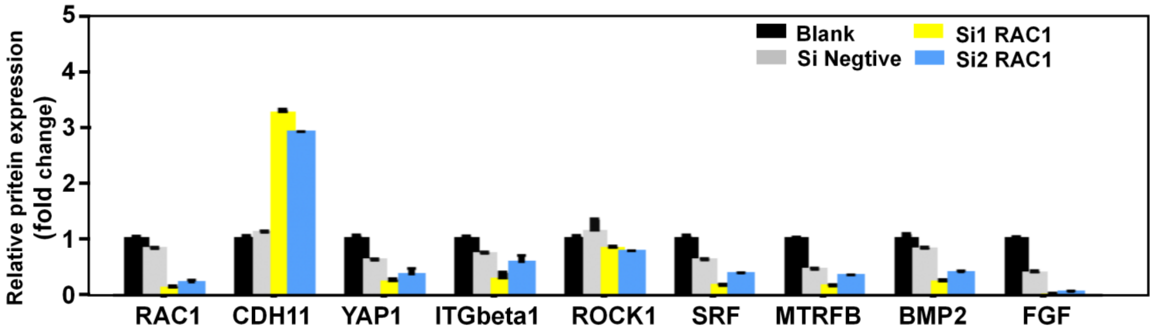

Figure S13. *RAC1 loss-of-function by RAC1 siRNA*.

siRNA inhibition of RAC1 decreased RAC1 downstream gene expression.

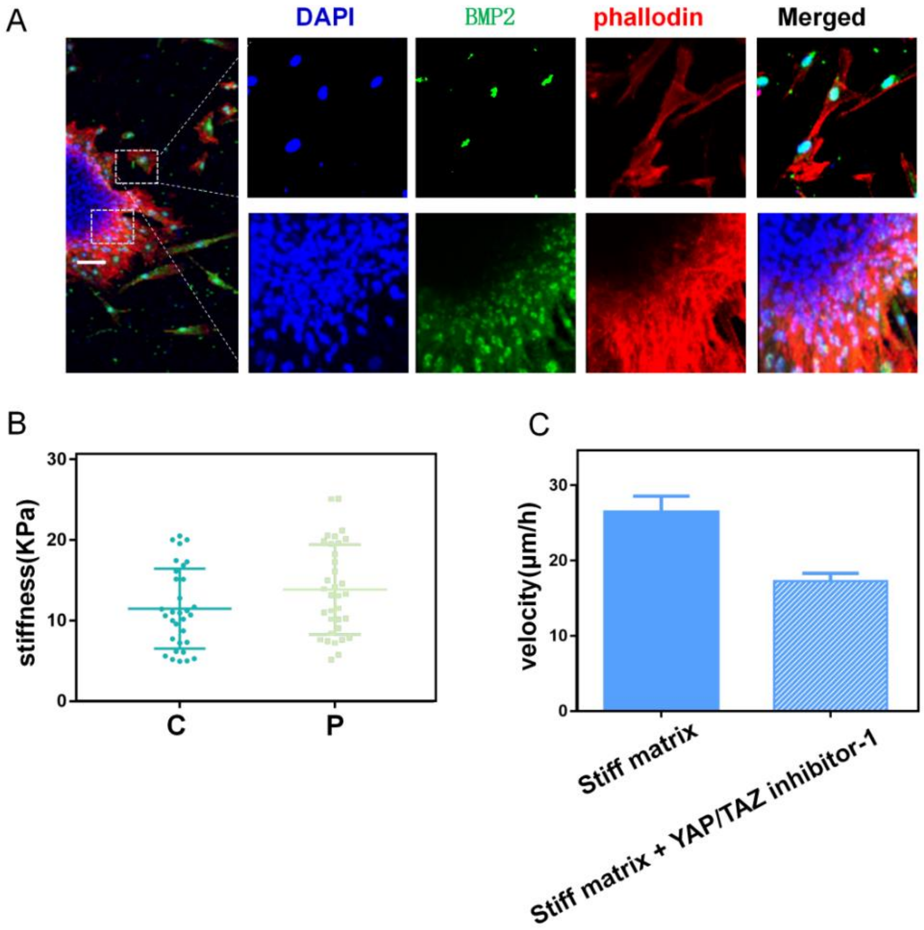

Figure S14. YAP1 loss-of-function by YAP/TAZ inhibitor-1 treatment.

The inhibited activation of YAP not only reduced the expression of the differentiation markers (*A*), but also significantly decreased cell stiffness (*B*) and spreading velocity (*C*). Scale bars, 100 μm in wide-fields; 25 μm in insets.
